# Potential Cardiovascular and Metabolic Beneficial Effects of ω-3 PUFA in Male Obesity Secondary Hypogonadism Syndrome

**DOI:** 10.3390/nu12092519

**Published:** 2020-08-20

**Authors:** Annalisa Noce, Giulia Marrone, Francesca Di Daniele, Manuela Di Lauro, Anna Pietroboni Zaitseva, Georgia Wilson Jones, Antonino De Lorenzo, Nicola Di Daniele

**Affiliations:** 1 UOC of Internal Medicine-Center of Hypertension and Nephrology Unit, Department of Systems Medicine, University of Rome Tor Vergata, 00133 Rome, Italy; francesca.didaniele@gmail.com (F.D.D.); dilauromanuela@gmail.com (M.D.L.); annapietroboni@icloud.com (A.P.Z.); georgia.wilson.jones@gmail.com (G.W.J.); didaniele@med.uniroma2.it (N.D.D.); 2 PhD School of Applied Medical, Surgical Sciences, University of Rome Tor Vergata, 00133 Rome, Italy; 3 Section of Clinical Nutrition and Nutrigenomic, Department of Biomedicine and Prevention, University of Rome Tor Vergata, 00133 Rome, Italy; delorenzo@uniroma2.it

**Keywords:** male obesity secondary hypogonadism (MOSH) syndrome, ω-3 PUFA, adipose tissue, body weight, testosterone

## Abstract

Long-chain ω-3 polyunsaturated fatty acids (PUFAs) are fundamental biocomponents of lipids and cell membranes. They are involved in the maintenance of cellular homeostasis and they are able to exert anti-inflammatory and cardioprotective actions. Thanks to their potential beneficial effects on the cardiovascular system, metabolic axis and body composition, we have examined their action in subjects affected by male obesity secondary hypogonadism (MOSH) syndrome. MOSH syndrome is characterized by the presence of obesity associated with the alteration of sexual and metabolic functions. Therefore, this review article aims to analyze scientific literature regarding the possible benefits of ω-3 PUFA administration in subjects affected by MOSH syndrome. We conclude that there are strong evidences supporting ω-3 PUFA administration and/or supplementation for the treatment and management of MOSH patients.

## 1. Introduction

It is truly fascinating to study how lifestyle modification can alter the course of a disease by modifying genetic expression and protein synthesis patterns. Thanks to modern epigenetics, researchers have found that changes in daily habits coupled with healthy nutrition can literally modulate our gene expression, in order to achieve better metabolic profiles and decrease the risk of developing an array of diseases [1]. Exploring the properties of natural compounds such as ω-3 polyunsaturated fatty acids (PUFAs) and how they can be optimally integrated in the diet is of paramount importance. Obesity represents a major public health burden and it can be defined as a pathological increase in weight and therefore in body mass index (BMI). 

PUFA ω-3 would seem to exert a cardioprotective role as they improve heart rate variability, a non-invasive marker of cardiac autonomic system function, with a subsequent reduction in the risk of sudden cardiac death and arrhythmias [2]. A further beneficial effect induced by PUFAs is linked to their anti-inflammatory capacity [3] and their ability to modulate the inflammatory response. Moreover, their effects in terms of improving body composition have also been recently demonstrated [4].

Obesity is defined as a condition characterized by a pathological increase in weight and therefore in body mass index (BMI). Its interpretation is based on weight status groupings, calculated by weight in kg divided by the square of the height in meters. A BMI exceeding 30 kg/m^2^ is indicative of obesity, as BMI rises, its values can be further subdivided into different classes correlating with different degrees of severity and cardiovascular disease (CVD) risk (class I between 30 and 34.9 kg/m^2^, class II between 35 and 39.9 kg/m^2^ and class III ≥40kg/m^2^) [5]. A BMI greater than 40 kg/m^2^ is defined as extreme, severe or morbid, whilst having a BMI between 25 and 30 kg/m^2^ is described as being in a state termed pre-obesity [6,7]. A sedentary lifestyle coupled with unhealthy eating habits, characterized by the excessive consumption of high energy foods, are the root of the growing prevalence of obesity worldwide. The mechanisms which have led to such a dramatic increase in the incidence and prevalence of obesity are complex and are intertwined with environmental and societal trends [8]. It is not uncommon nowadays to see the term obesity flanked by the term epidemic or even pandemic. This is due to the sheer statistics regarding obesity, which estimate that in 2016 there were 1.9 billion overweight adults worldwide [9,10]. Obesity is the pathophysiological state determined by weight and adipose excess, which is characterized by the alteration of body composition starting from peripheral tissues such as adipose tissue, liver and muscles [11]. These alterations lead to an increased risk of the onset of arterial hypertension, CVDs and other chronic non-communicable degenerative diseases (CNCDs), such as type 2 diabetes mellitus (T2DM), male obesity secondary hypogonadism (MOSH), respiratory diseases, cancer, chronic kidney disease and psychopathological alterations that negatively impact on both quality of life and longevity [12,13,14,15].

In obese men, MOSH syndrome leads to a plethora of symptoms such as impaired fertility and sexual function, deficient bone mineralization, altered fat metabolism and body composition and the deterioration of muscle mass [16]. Epidemiological data obtained by population studies state that the prevalence of MOSH syndrome is above 45–57.5% of male obese subjects and it correlates with high-rate morbidity and mortality [17,18].

In this review article we analyzed the possible beneficial effects of ω-3 PUFA on clinical signs and symptoms of MOSH syndrome.

## 2. Methods 

Current literature investigating the possible positive impact of ω-3 PUFA consumption on MOSH syndrome is analyzed and contextualized in this review. Specifically, research has been conducted on Medline (Pubmed) and Scopus. Such a review article analyzes studies (both in vivo and in vitro studies) published up to June 2020.

## 3. Structure, Metabolic Pathways and Dietary Sources of PUFA

Fatty acids (FAs) are fundamental biocomponents of lipids and cell membranes. They are made up by a hydrocarbon backbone and a carboxylic head group. FAs are classified according to the length of the hydrocarbon backbone (generally 12 to 24 carbon atoms long), and according to the presence and the number of double bonds. We can distinguish between saturated fatty acids (SFAs), which are characterized by the absence of double bonds, monounsaturated fatty acids (MUFAs), which only have one double bond and PUFAs, in which more than one double bond may be found. FAs can be further classified according to the position of the first double bond compared to carbon ω (the furthest carbon from the carboxylic group), forming two classes: ω-3 and ω-6 PUFA [19,20]. 

The human body can produce almost all fatty acids, except α-linolenic acid (ALA, C18:3 ω-3) and linolenic acid (LA, C18:2 ω-6) which are precursors of ω-3 and ω-6 PUFAs. These are termed “essential fatty acids” because they can only be obtained through diet [21]. Through endogenous conversion (elongation and desaturation) the organism is capable of synthesizing longer-chain counterparts such as eicosapentaenoic acid (EPA) and docosahexaenoic acid (DHA) in the ω-3 family, and γ-linolenic acid (GLA), dihomo-γ-linolenic acid (DGLA) and arachidonic acid (AA) in the ω-6 family [20,22].

Long-chain ω-3 PUFAs and long-chain ω-6 PUFAs are precursors of molecules with important biological activity called eicosanoids such as prostaglandins (PG), thromboxanes (Tx), leukotrienes (LTS), lipoxins (LXS) and resolvins. Depending on which precursor family they belong to, PUFAs can perform different biological functions. In fact, while ω-3 PUFAs carry out an anti-inflammatory function, ω-6 PUFAs elicit a proinflammatory function. 

The ω-3 and ω-6 long-chain PUFAs compete to bind enzymes such as cyclooxygenase, lipoxygenase and epoxygenases, which are responsible for the release of inflammatory mediators. Thus, the equilibrium between ω-3 and ω-6 PUFA intracellular concentrations is fundamental for the maintenance of cellular homeostasis and cardiovascular (CV) protection [20,23,24]. In order for them to perform their correct biological actions, it is necessary to have a balanced PUFA intake. Recent studies suggest that an ideal ratio between ω-6/ω-3 is between 1:1 and 1:5, whilst the actual intake ratio in Western countries is of 15:1–16.7:1. Therefore, it appears necessary to maintain an adequate and balanced intake of ω-6/ω-3 in order to prevent CVD onset [25,26,27].

Regarding main food sources, PUFAs are present as precursors (ALA and LA) in plant-based products and as derivatives (EPA, DHA, AA) in meat (Table 1 and Table 2). Fish is the main source of long-chain ω-3 PUFAs, including EPA, DHA and docosapentaenoic acid (DPA), while ALA is a plant and ω-3 PUFAs are mainly found in seeds and nuts and their oils. Plant sources of ω-3 PUFAs cannot currently be considered as a replacement for seafood-derived ω-3 PUFAs. This suggests that ω-3 PUFAs, derived from different sources, have their own specific effects. Therefore, it appears necessary to have a varied and balanced diet [20,27].

ω-3 PUFAs are also known as “vitamin F”, not only are they needed for basic cellular functions such as cell signaling, membrane fluidity and structural integrity, but also for nervous system regulation [28,29]. They have a role in regulating blood pressure, clotting, glucose metabolism and inflammation [28]. Moreover, they have been related to be preventative in the occurrence of CVevents and to slow down the progression of CVDs. These concepts will be further explored in the following section [30].

## 4. Male Obesity Secondary Hypogonadism (MOSH) Syndrome Definition

MOSH syndrome is a clinical condition found in obese middle-aged men and epidemiological reports assert that in the last 10 years its prevalence has enhanced, even if it is currently an underestimated and underdiagnosed condition [31].

**Figure 1:**
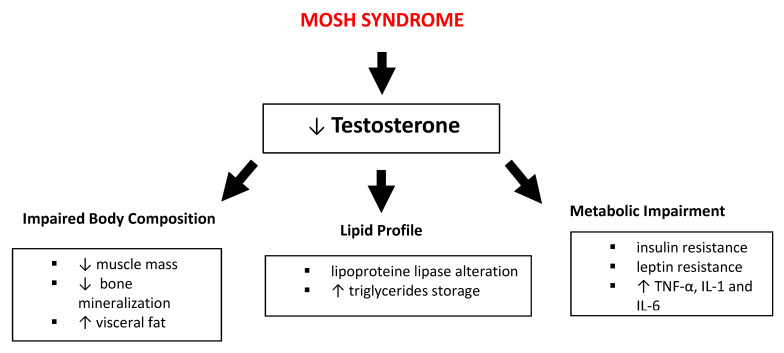
Impact of male obesity secondary hypogonadism (MOSH) syndrome on body composition, lipid profile and metabolic pathways. Abbreviations: TNF-α, tumor necrosis factor-α; IL, interleukin; ↑: increase; ↓: decrease.

In MOSH syndrome, obesity corroborates hypogonadism to give rise to reduced levels of testosterone (T). This reduction is due to the alteration of metabolic patterns such as lipid metabolism, chronic inflammation and insulin resistance (Figure 1) [32]. 

The pathophysiological mechanisms linking obesity with hypogonadism are complex and multifactorial [32]. Obese male subjects show a significant reduction of T levels caused by an increase of aromatase enzymes levels, released by the adipose tissue and enhanced by estrogen hormones [33], coupled with the negative feedback produced by the estrogen on the hypothalamic-pituitary axis, this is another factor decreasing the circulating T levels. Such pattern affects the lipid profile through the alteration of lipoprotein lipase presence on adipocytes and increase triglycerides (TG) storage, leading to an increase in visceral adipose deposition and total body fat. These alterations are considered particularly harmful and are highly associated with CV disease risk [33]. Moreover, these lipid profile alterations create a sort of self-perpetuating cycle between obesity and hypogonadism.

The hypertrophy of adipose tissue, characteristic of obese subjects, leads to the lowering of T levels. Metabolic impairment caused by body fat enhancement is responsible for insulin and leptin resistance, and for the increase of pro-inflammatory cytokines (such as Tumor Necrosis Factor-α - TNF-α, interleukins 1 and 6 - IL-1,IL-6) which influence hypothalamic function, in particular decreasing kisspeptin signaling [34]. Such a decrease entails the reduction of gonadotropin-releasing hormone (GnRH), which in turn decreases luteinizing hormone (LH) and follicle-stimulating hormone (FSH) secretion by anterior pituitary gonadotrophs, resulting in a T reduction and in the alteration of fertility [35]. 

Subjects affected by MOSH are often characterized by reduced osseous mineral density, which can be explained by the T deficiency that is strongly involved in the modulation of bone mineralization, as T is implicated in the regulation of the proliferation and differentiation of osteoblasts [16].

T induces skeletal muscle hypertrophy through numerous mechanisms including its effects in modulating pluripotent mesenchymal cell engagement. Studies have shown that elevated T levels are associated with an increase in the size of motor neurons [36]. Therefore, in subjects with MOSH, the reduction of T levels can lead to a reduction in muscle mass.

MOSH syndrome is potentially reversible. Its treatment, in addition to exogenous T administration, includes lifestyle changes such as diet therapy and physical activity aimed to reduce obesity [16,37,38]. 

### 4.1. Role of PUFA in Cardiovascular Disease

In the last few years, the role of ω-3 PUFAs has been widely debated within the scientific and medical communities in virtue of the possible role they may play in contrasting CV diseases (Table 3). 

On the one hand observational studies reported an inverse association between CV diseases and dietary intake or plasma concentrations of ω-3 PUFAs (primarily EPA and DHA), suggesting that their supplementation might exert cardio protective effects, on the other hand successive clinical trials and metanalyses have speculated the absence of true benefits induced by ω-3 PUFA consumption on the CV system [39,40,41,42]. This discrepancy may be justified by the multiple variables that influence CV diseases which may lead to contrasting results. These variables render CV diseases quite heterogeneous, resulting in different responses to ω-3 PUFA treatment. We must take into consideration that this kind of treatment does not carry out the action of a “pharmaceutical” drug, but rather acts by producing a modulatory effect on the subject’s metabolism which can be more or less susceptible to a response, depending not only on the degree and type of pathological involvement but also on the subject’s genetic susceptibility. This renders the task even more articulated, particularly as an individual’s genetic susceptibility is determined by the genotype and by environmental and epigenetic changes. Even if the debate on ω-3 PUFAs is currently unresolved, it is worth underlining that their consumption has never been associated with deleterious effects on health and therefore their use can either induce positive CV effects, or in the worst case scenario, can induce a neutral effect [43]. For such reason, the following section will comment on the possible beneficial health effects induced by PUFA consumption in subjects with an elevated CV risk and in patients affected by MOSH syndrome. The cardioprotective role of ω-3 PUFAs was hypothesized for the first time in the 1950s in the Eskimo population, which presented elevated levels of plasma cholesterol but an exiguous CV mortality rate [44]. Successively, such observation was also made in the Japanese and Icelandic populations, in which there was an evidently low mortality from CV pathologies compared to Western populations [45,46]. This cardio protective effect was attributed to eating habits, in particular to elevated fish consumption. Further epidemiological studies confirmed this correlation and described the cardioprotective effects induced by ω-3 PUFA consumption [47]. In light of the data published by two large clinical randomized trials, the American Heart Association (AHA) in 2002 suggested the consumption of 1g/day EPA+DHA in patients with coronary artery disease in virtue of their cardioprotective potential [48,49,50]. Successively, the Gruppo Italiano per lo Studio della Streptochinasi nell’Infarto (GISSI) [50,51] and Diet And Reinfarction Trial (DART) [48] studies have demonstrated a reduction in CV risk following treatment with ω-3 PUFAs, representing the milestones of clinical recommendations for ω-3 PUFA treatment in cardiopathic subjects since it was observed that the benefits outweighed any possible side effect related to their consumption [52,53]. The main cardio protective effects induced by ω-3 PUFA consumption are achieved through actions such as the reduction of plasma TG and of chronic low-grade inflammatory status, an improvement of endothelial function, cardiac functional remodeling and of cardiac contractility [51,54,55]. An in vitro study conducted in bovine aortic endothelial cells demonstrated that treatment with adiponectin is able to increase nitric oxide (NO) production by 3-fold in endothelial cells. This action is due to the phosphorylation of endothelial-nitric oxide synthase (e-NOS) by phosphatidylinositol 3-kinase-dependent pathways [56]. In 2002, the AHA affirmed that a dose between 2 and 4 g/day of ω-3 PUFA was able to treat hypertriglyceridemia [57]. In the wake of this finding, one of the principal studies, aimed at underlining an improvement in plasma TG, was conducted by Harris et al. [58] These authors observed a dose-dependent plasma TG reduction after ω-3 PUFA administration, especially in subjects who presented basal TG levels >500 mg/dL [58]. This was confirmed in subsequent clinical trials performed on subjects presenting very high triglyceride (VHT) levels (TG > 500 mg/dL) and high triglyceride (HT) levels (TG between 200 and 499 mg/dL). Results showed a 30% reduction in plasma TG in the VHT group and a reduction between 20 and 30% in the HT group following the consumption of 4 g/day of ω-3, confirming that the reduction in percentage of TG correlated with their plasma levels before treatment [59,60,61]. ω-3 PUFAs are able to contrast chronic inflammation via the reduction of macrophage-monocyte adhesion, caused by oxidized low-density lipoprotein (LDL) to the endothelial lining of the coronary vessels. This effect is coupled with the increased expression of e-NOS induced by DHA, with a consequent increase in NO release and therefore, vasodilation [62]. DHA is also able to modulate endothelial function by inducing the transcription of the gene coding for the proinflammatory cytokine TNF-α, and the inhibition of the pathway generated by nuclear factor kappa-light-chain-enhancer of activated B cells (NF-κB), which causes a reduction in vascular cell adhesion molecule-1 (VCAM-1) [63]. Therefore, the actions carried out by DHA at the endothelial level suggest its vasoprotective role. 

Moreover, ω-3 PUFAs induce the suppression of thromboxane A_2_ (a factor responsible for platelet aggregation, vasoconstriction and fibrinogen reduction) synthesis, and favor the synthesis of thromboxane A_3_ [64,65,66]. In this context, animal models highlighted that EPA consumption also plays a role in stabilizing the atheromatous plaque [67].

EPA and DHA inhibit a series of processes linked to inflammation, such as leukocyte chemotaxis, adhesion interactions between leukocytes and the endothelium, eicosanoid production and T cell reactivity [68]. Finally, an increase in EPA and DHA availability modifies the equilibrium between ω-3 and ω-6 PUFAs, favoring anti-inflammatory eicosanoid synthesis [69].

ω-3 PUFA consumption is associated with a better vascular function, playing a protective role in atherosclerosis, in which endothelial dysfunction is at the basis of the pathogenic process [62,70]. ω-3 PUFAs improve arterial wall rigidity [71] and it was observed that their supplementation induces a reduction in endothelial damage biomarkers such as E-selectin [72].

ω-3 PUFA supplementation was also associated with the reduction of heart rate at rest [73,74], the reduction of systolic and diastolic blood [75,76], and the increase in early and late left ventricular ejection fraction [77]. 

ω-3 PUFA treatment can lead to a reduction in hospitalization and CV mortality incidence [52]. Finally, the study OMEGA-REMODEL has demonstrated a reduction in cardiac remodeling and fibrosis markers in patients with acute myocardial infarction (AMI), following a supplementation of ω-3 PUFAs (4 g/day) in the diet [78]. It is hypothesized that this beneficial effect is correlated with the reduction of macrophage activation and with the inhibition of galectin-3 (Gal-3), a factor which reflects cardiac function impairment and remodeling [79]. In an elderly population in which subjects had recently undergone an AMI, there were significant inverse correlations between ω-3 PUFA content in serum phospholipids and serum levels of Gal-3, confirming the beneficial effects of ω-3 PUFAs on cardiac remodeling [79].

### 4.2. Impact of PUFA Consumption on Body Weight

The Mediterranean diet is known to provide a balanced supply of PUFAs [14]. In vivo studies have demonstrated that the consumption ω-3 FA is correlated with the improvement of body composition. Specifically, it is observed that there is a reduction in adipose tissue thanks to the interactions with metabolic pathways, including the glucose one [80]. A meta-analysis conducted in 2014 [4] has explored the relationship between the consumption of long-chain ω-3 PUFAs and body composition in Caucasian subjects (Table 4). The study examined 934 subjects who were getting long-chain ω-3 PUFAs from fish or from supplements. The authors have found statistically significant variations comparing results obtained between the study group and healthy subjects. The examined parameters were: body weight; BMI; fat mass (FM) %; and waist circumference (WC). Moreover, the authors have also investigated the possible gender effect tied to the consumption of long-chain ω-3 PUFAs, highlighting that in male subjects the WC diminished significantly more than in females. 

There is considerable evidence showing that, at the cellular level, PUFAs are potent transcription regulators of genes involved in lipid metabolism. In fact, PUFAs have an important role in the inhibition of genes involved in lipogenesis, and in the promotion of genes involved in lipid oxidation [81]. Other than being prone to rapid oxidation and peroxidation, PUFAs are able to favor the synthesis of proteins involved in detoxification processes that counteract oxidative stress [82]. A study by Di Nunzio et al. [83] has shed light on the antioxidant and pro-oxidant properties of different PUFAs. The authors have demonstrated that only DHA is able to diminish susceptibility to hydrogen peroxide, which stimulates the transcription and the activation of the peroxisome proliferator-activated receptor α (PPARα). PPARα is able to favor the activity of antioxidant enzymes, such as Catalase- CAT and superoxide dismutase- SOD [83]. Therefore, the consumption of PUFAs, specifically DHA, allows an adequate antioxidant protection at the cellular level if the ω-3/ω-6 at 1:5 ratio is followed [84].

The enhancement in lipid oxidation, and the increased use of lipids as an energy source, can translate into a reduction in FM. In fact, some studies demonstrate that increased PUFA intake is associated with substantial FM loss, especially in the abdominal region [85]. 

Couet et al. have examined a population of lean and healthy individuals who were administered 6 g/day of visible fat for 3 weeks followed by a wash-out period lasting 10–12 weeks, followed in turn by the administration of 6 g/day of fish oil for 3 weeks. The authors have reported a statistically significant reduction in FM, whilst body weight was maintained [86].

A study by Huang et al. [87] has examined the possible genetic–dietary interactions in a population of 24,357 subjects. The authors have analyzed all known 77 single-nucleotide polymorphisms (SNPs) correlated with BMI. The data showed that consumption of fish-derived long-chain ω-3 are able to modulate gene expression related to weight gain and BMI modifications. In fact, long-chain ω-3 PUFAs were able to modify the genetic associations that determine adipose tissue accumulation in various body regions [88]. Therefore, the consumption of long-chain ω-3 PUFAs plays an important role in phenotype manifestation, modulating the expression of weight regulatory genes. 

The notion that adipose tissue is simply an inert tissue that stores fat has become obsolete. On the contrary, it is now recognized as a metabolically active endocrine organ, which has the capacity to synthesize biological mediators called adipocytokines, which regulate the body’s metabolic status and influence homeostasis [89]. Adipose tissue is not solely comprised of adipocytes, but also of blood vessels and stroma, which contain the precursor cells. It is also useful to distinguish white adipose tissue (WAT) from brown adipose tissue (BAT). WAT is made up of unilocular adipocytes and is better suited for storage, while BAT adipocytes are multilocular, contain copious amounts of mitochondria and are involved in thermogenesis [90]. Diet-induced thermogenesis is a metabolic process linked with energy expenditure following the ingestion of various macronutrients (such as carbohydrates, proteins, fats and alcohol). A study by Casas-Agustench et al. has examined a population of 29 healthy males and compares the thermogenic effects induced by three isocaloric meals: the first contained high levels of PUFAs from walnuts, the second contained high levels of MUFAs from olive oil and the third contained high levels of fat from dairy products. Thermogenesis induced 5 hours after the first meal was 28% greater than the one induced by the third meal. Therefore, the quality of fats can influence the thermogenic response, even if the properties which influence lipid substrate oxidation are still not known [91].

A further feature of obese subjects is the low-grade chronic inflammatory state. The postulation that obesity is inherently linked to the latter goes hand in hand with the notion that adipose tissue in an obese individual undergoes compelling alterations in both composition and function, a process named “adipose tissue remodeling” [92]. The inflammatory status is characterized by pro-inflammatory molecules such as TNF-α, interleukin (IL)-1*β*, IL-6, IL-8, transforming growth factor-*β*, nerve growth factor and acute phase response molecules such as plasminogen activator inhibitor-1, haptoglobin; serum amyloid A, has been recognized as a driver of metabolic disease in obese subjects [93]. Therefore, a reduction of the low-grade chronic inflammatory status, consequent to a decrease of body weight, would lead to an improvement in the clinical conditions of MOSH syndrome.

A study by Lund et al. [94], other than attaining positive results regarding BMI, WC and hip circumference (HC) reduction following PUFA consumption, has highlighted an inverse correlation between ALA ω -3 consumption and levels of macrophage inflammatory protein (MIP)-1α. The latter is a chemokine which is overexpressed in obese subjects who present abdominal visceral fat accumulation. Therefore, PUFAs are able to act beneficially on MIP-1α levels, and therefore on central adiposity.

### 4.3. PUFA and Metabolic Axis

Long-chain ω-3 PUFAs are able to regulate numerous metabolic mechanisms apt to contrast weight gain. They enable better control of the hunger and satiety mechanism and allow better perfusion of metabolically active tissues (such as skeletal muscle) through the modulation of gene expression. They also induce fatty acid oxidation and can cause an increase in energy expenditure associated with a reduction in fat deposits [95]. 

Several studies suggest that long-chain ω-3 PUFAs can suppress appetite and regulate thermogenesis by inducing an increase in blood concentration of adipocyte hormones such as leptin and adiponectin [80,96,97] (Table 5). Leptin was the first hormone to be recognized for having a regulatory action at the hypothalamic level [98]. Its principal function is to control food-intake, undertaking an anorexigenic effect, however, it can also regulate energy expenditure and body weight [99]. Leptin acts upon the metabolism and food consumption, reducing appetite and increasing energy expenditure [100]. The expression and release of this hormone are positively correlated with the amount of fat mass and adipocyte dimension, and they are stimulated by hormones such as cortisol and insulin [101].

Different studies report that the reduction in leptin plasma concentration represents a short-term adaptation to the mechanism of hunger or fasting and therefore, in response to diet-induced weight loss, the levels of leptin decrease significantly [102,103]. In normal weight subjects, leptin is released into circulation and acts through hypothalamic and extra-hypothalamic brain receptors (arcuate nucleus and dorsomedial hypothalamus, respectively), inhibiting hunger and increasing thermogenesis following food intake. Moreover, in non-obese subjects, leptin acts through hypothalamic receptors, inhibiting the hunger mechanism and increasing thermogenesis during the fasting period. Decreased leptin levels provoke a reduction in central sympathetic nervous outflow and mobilize stored adipose tissue through glucocorticoid stimulation [104]. Whereas in obese subjects, even if plasma leptin concentration seems to be increased, it does not decrease food consumption and increase energy expenditure. Such a phenomenon suggests that obese subjects become leptin-resistant as reported by different authors since the 1990s [98,104]. The “leptin resistance hypothesis” was demonstrated by Enriori et al. and observed an attenuation of the phosphorylation of signal transducer and activator of transcription 3 (STAT3) in obese mice, which is a crucial factor for the action of leptin on the hypothalamic arcuate nucleus [105]. 

Hyperleptinemia is also associated with an increased production and release into the bloodstream of pro-inflammatory cytokines (such as TNF-α, C- reactive protein- CRP, etc.) [106,107] and to an increase of platelet aggregation and thrombosis [108]. Thus, the persistent condition of hyperleptinemia could play an unfavorable role in different organs and systems such as the CV system.

A study by Pérez-Matute et al. [109] investigated the potential anti-obesogenic and insulin-sensitizing properties associated with long-chain ω-3 PUFA consumption in an animal model, this was done by feeding the animals two different dietary regimens for the duration of 5 weeks. The control group was administered a standard laboratory diet, whilst the study group was administered a fat-rich hyperenergetic diet. These groups were further divided into two subgroups, differentiated by whether or not they were administered EPA. Results showed that EPA consumption during a fat-rich hyperenergetic diet is able to restrain weight gain and consequently leads to an increase in fat mass. This effect could be correlated with an increase in leptin levels, which causes reduced hunger. Another finding shows that the group consuming a fat-rich hyperenergetic diet and EPA supplementation showed significant weight loss, greater than the standard laboratory diet and EPA supplementation group. It can be speculated that the metabolic effects related to a fat-rich hyperenergetic diet could be correlated to its bromatological composition.

Adiponectin is a protein which regulates the endocrine functions of adipocytes, which perform autocrine and paracrine functions. Adiponectin seems to improve lipid storage, contrasting ectopic deposition of lipids [110] favoring healthy adipose tissue composition. Moreover, it can regulate energy homeostasis by modulating lipid and the glucose metabolism as well as fatty acid oxidation. A study highlighted that adiponectin is able to ameliorate insulin sensitivity in the liver and in skeletal muscles, regulating healthy adipose tissue expansion [111,112]. A study was conducted by Dimiter [113] to investigate the relationship between ω-3 PUFA consumption and circulating adiponectin levels on 35 subjects with metabolic syndrome. The subjects were subdivided into two groups: one was treated with ω-3 PUFA supplements and the control group was given a placebo for a period of three months. The results showed that the treated group demonstrated a statistically significant increase in plasma adiponectin and high-density lipoprotein (HDL) cholesterol, with a concomitant decrease in TGs, Homeostatic model assessment - insulin resistance (HOMA-IR) and CRP. These findings highlighted that supplementation with ω-3 PUFAs can contribute to a bettering of the clinical profile of metabolic syndrome patients by reducing inflammation, improving dyslipidemia and endocrine function through adiponectin-dependent mechanisms.

Long-chain ω-3 PUFAs can alter gene expression in skeletal muscle, suppressing catabolic pathways and upregulating anabolic ones. These mechanisms attenuate muscular mass loss while maintaining muscular functionality and metabolic rate [95]. The restriction of energetic intake results in efficacious fat mass reduction; however, it can often cause the loss of fat-free mass and skeletal muscle. This may negatively impact on physical performance and cause a reduction in metabolic rate by reducing lipid oxidation capacity [114]. The principal pathway responsible for muscle catabolism during energetic intake restriction is the ubiquitin-proteasome pathway [115]. EPA is able to inhibit the activity of such a pathway during periods of severe energy intake restriction. In this context, long-chain ω-3 PUFAs can augment the activation of the Protein kinase B (Akt)—Mammalian target of rapamycin (mTOR)—the Ribosomal protein S6 kinase beta-1 (S6K1) anabolic pathway in skeletal muscle-promoting anti-catabolites and anabolites [116]. In a study by Howe et al. [117], long-chain ω-3 PUFAs were able to attenuate muscle mass loss during an energy restriction diet. Moreover, an improvement of lean mass and energy balance was observed [95,118]. Successively, the same authors have observed an increase in lean mass percentage, suggesting a direct relationship between the consumption of ω-3 PUFAs and lean mass improvement [117].

## 5. Summary and Future Perspectives

In conclusion, there seems to be evidence that ω-3 PUFA consumption may be clinically beneficial in the treatment and clinical management of MOSH patients. 

The ability of ω-3 PUFAs to act on some pathological aspects of MOSH, such as obesity, inflammation, metabolic and cardiovascular disorders, coupled with their optimal safety profile, leads us to the postulation that ω-3 PUFA assumption could be a valuable tool in ameliorating the clinical manifestations of MOSH syndrome.

In pursuance to assess this conclusion in a definitive manner and in order to define the most advantageous dosage, randomized clinical trials on a large population sample are required. Moreover, it would be interesting and useful to conduct experimental studies exploring the possible effects of ω-3 PUFA consumption on hormone profile, on the sexual sphere (T concentrations) and on body composition.

## Figures and Tables

**Table 1:** Main dietary sources of ω-3 fatty acids.

Ω-3 Series	Foods
ALA	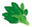 Green leafy vegetables, nuts, soybeans, flaxseed 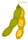
EPA	Fish oil(herring, salmon, sardine, cod liver), Fish(caviar black and red, herring, salmon)
DHA	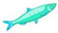 Fish oil, herring, caviar black and red, salmon 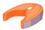

Abbreviations: ALA, α-linoleic acid; DHA, docosahexaenoic acid; EPA, eicosapentaenoic acid.

**Table 2:** Main dietary sources of ω-6 fatty acids.

Ω-6 Series	Foods
LA	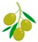 Olive oil, seed oil 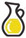
GLA	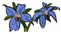 Borage oil, black currant oil 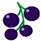
DGLA	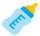 Human milk
AA	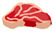 Meat, dairy, shelfish, human milk 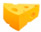

Abbreviations: AA, arachidonic acid; LA, linoleic acid; DGLA, dihomo-γ-linolenic acid; GLA, γ-linolenic acid.

**Table 3:** Studies on polyunsaturated fatty acids (PUFAs) and cardiovascular disease.

Type of the Study	Reference	Year	Type of Intervention	Primary Outcome	Conclusions
Animal	Wang, T.M. et al. [63]	2011	40 ApoE (-/-) knockout mice randomized into 5 groups: 1 control group fed normal chow diet, 4 groups fed chow diet supplemented with 200 mg/kg/day of (i) DHA, (ii) EPA, (iii) LA or (iv) AA, for 10 weeks.	DHA supplementation reduced the expression of VCAM-1 in a dose-dependent manner in TNF-α -activated aortic endothelial cells.	DHA supplementation acts at endothelial level by exercising a vasoprotective role.
	Matsumoto, M. et al. [67]	2008	12 ApoE-deficient mice were fed with Western diet and randomized into two groups: (i) 5% EPA supplementation (ii) without EPA supplementation, for 13 weeks.	The EPA supplementation group showed a reduction in the development of atherosclerotic lesions. Lesions presented a great amount collagen and smooth muscle cells and lower amount of macrophages.	EPA has anti-inflammatory and stabilizing effects on the atherosclerotic plaque.
Human	Rhee J.J. et al. [42]	2017	Prospective cohort study on a total of 39,876 women aged ≥45 years without CV diseases subjected to questionnaires on food frequency.	During the follow-up (1993–2014) period there were no associations between the consumption of fish and ω-3 PUFA and CV disease.	The consumption of ω-3 PUFA does not reduce CV risk.
	GISSI-Prevenzione Investigators [51]	1999	Controlled study conducted on 11,324 patients who survived myocardial infarction. They were randomized into 4 groups taking: (i) ω-3 PUFA supplements (1 g per day), (ii) vitamin E (300 mg per day), (iii) both or (iv) neither, for 3–5 years.	The groups with ω-3 PUFA supplementation and with ω-3 PUFA + vitamin E supplementation, presented a reduced risk of death due to CV causes, equally.	Supplementation with ω-3 PUFA reduces the risk of CV mortality.
	Bays, H.E. et al. [59]	2011	Double blind controlled study conducted on 229 subjects with highly elevated blood levels of triglycerides (≥500 mg/dL), which were randomized into 3 groups: (i) 4 g/day supplementation of EPA ethyl ester (ii) 2 g/day supplementation of EPA ethyl ester or (iii) placebo, for 12 weeks.	The supplementation of 4 g/day EPA ethyl ester reduced triglyceride levels by 33.1% whereas 2 g/day supplementation led to 19.2% reduction, both compared to placebo.	ω-3 PUFA supplementation can be useful to counteract hypertriglyceridemia.
	Kastelein, J.J. et al. [60]	2014	Double-blind controlled study conducted on subjects with highly elevated blood levels of triglycerides (≥500 mg/dL) which were randomized into 4 groups: (i) control group (4 g/day of olive oil), (ii) 2g/day of ω-3 PUFA, (iii) 3 g/day of ω-3 PUFA, (iv) 4 g/day of ω-3 PUFA, for 12 weeks in combination with a nutrition education program.	25.9%, 25.5% and 30.9% reduction in blood triglycerides with supplementation of 2, 3 and 4 g/day of ω-3 PUFA respectively, compared to placebo group.	ω-3 PUFA supplementation can be used in lowering hypertriglyceridemia.
	Maki, K.C. et al. [61]	2013	647 patients with triglyceride values ≥200 mg/dL and <500 mg/dL were randomized to 6 weeks of treatment with control capsules (4 g/d of olive oil), 2 g/day ω-3 PUFA + 2 g/day olive oil or 4 g/d of ω-3 PUFA.	14.6% and 20.6% reduction in triglyceride levels and 3.9% and 6.9% reduction in non-HDL cholesterol levels with supplementation of 2 g/day and 4 g/d respectively ω-3 PUFA.	ω-3 PUFA supplementation can be useful in the control of dyslipidemias.
	Casanova, M.A. et al. [71]	2017	29 adults with hypertriglyceridemia were divided into: high risk CV patients and low risk CV patients, randomized to take ω -3 PUFA (1800 mg/day) or ciprofibrate (100 mg/day) for 12 weeks.	In high-risk patients, pulse wave velocity was reduced and flow-mediated dilation was increased by ω-3 PUFA.	ω -3 PUFA improved arterial stiffness and endothelial function.
	Huang, F. et al. [72]	2019	69 normal weight adolescents and 70 obese adolescents with hypertriglyceridemia were treated with a lifestyle intervention and randomized for ω -3 PUFA or placebo supplementation for 12 weeks.	After 12 weeks of omega-3 supplementation, adolescents showed a significant reduction in triglycerides, leptin, RBP4, ADMA and E-selectin compared to the placebo group and compared to lifestyle intervention alone.	ω-3 PUFA supplementation associated with a healthy lifestyle improves dyslipidemia, insulin resistance and endothelial dysfunction.
	Rantanen, J.M. et al. [73]	2018	Randomized, double-blind, controlled trial of 112 chronic dialysis patients from Denmark randomized for daily supplementation of 2 g marine ω-3 PUFA or control group, for three months.	In the group with daily supplementation with ω-3 PUFA there was a reduction in heart rate of 2.5 beats per minute, evaluated by 48-h Holter monitoring.	ω-3 PUFA could contribute to vagal modulation that could be protective against malignant ventricular arrhythmias.
	Sagara, M. et al. [75]	2011	38 men with arterial hypertension and/or hypercholesterolemia were randomized for a five-week dietary supplement with 2 g of DHA vs active placebo (1 g of olive oil).	Significant reduction in systolic and diastolic blood pressure, heart rate and HDL-C increase in the group with supplementation of DHA.	DHA supplementation can reduce coronary heart disease risk factors.
	Lee, J.B. et al. [76]	2019	Randomized double-blind study of 86 healthy young men and women to evaluate the effects of oral supplementation of 3 g/day of (i) EPA, (ii) DHA or (iii) olive oil for 12 weeks.	Reduction of systolic and diastolic BP at rest after DHA and olive oil supplementation compared to EPA; DHA supplementation enhances peripheral vasoconstrictor outflow.	DHA supplementation may represent a valid support for patients with high chronic BP.
	Ghio, S. et al. [77]	2010	608 patients with chronic symptomatic heart failure were randomized to take (i) ω-3 PUFA or (ii) placebo supplementation. Echocardiography was performed at baseline and after 1, 2 and 3 years.	Left ventricular ejection fraction increase after ω-3 PUFA supplementation of 8.1% at 1 year, 11.1% at 2 years and 11.5% at 3 years.	ω-3 PUFA supplementation can significantly improve left ventricular ejection fraction in patients with symptomatic heart failure.
	Tavazzi, L. et al. [52]	2008	Controlled, double-blind study in patients with chronic heart failure randomized for the supplementation of 1 g/day of (i) ω-3 PUFA or (ii) placebo, followed for a median of 3.9 years.	57% of patients in the ω-3 PUFA supplement group, compared to 59% in the placebo group, died or were hospitalized for CV reasons.	ω-3 PUFA supplementation may provide a small benefit in terms of mortality and hospitalization for CV reasons in heart failure patients.
	Heydari, B. et al. [78]	2016	Controlled, double-blind study on 358 patients after acute MI, randomized for (i) ω-3 PUFA supplementation (4 g/die) or (ii) placebo and underwent baseline assessment by CMR 4-28 days after MI, with 6 months follow-up.	After 6 months of PUFA treatment, the follow-up CMR revealed a significant reduction in left ventricular end-systolic volume indexed and myocardial extra-cellular volume fraction and ST2, fibrosis marker, compared to placebo.	PUFA have an important effect on phenotypes of myocardial tissue after MI.
	Laake, L. et al. [79]	2017	Evaluation of the relationship between serum level of marine PUFA ω-3 and ω-6 and biomarkers of fibrosis and cardiac remodeling (ST2 and Galectin-3) in 299 elderly patients 2–8 weeks after acute MI.	Galectin-3 levels were inversely related to EPA and DHA and positively related to the ω -6/ω -3 ratio.	ω-3 PUFA display positive effect on cardiac remodeling in acute MI elderly patients.

Abbreviations: AA, arachidonic acid; ADMA, asymmetric dimethylarginine; ApoE, apolipoprotein E; BP, blood pressure; CMR, Cardiac Magnetic Resonance; CV, Cardiovascular; DHA, docosahexaenoic acid; EPA, eicosapentaenoic acid; HDL, high-density lipoprotein; LA, linoleic acid; MI, myocardial infarction; PUFA, polyunsaturated fatty acids; RBP4, retinol binding protein 4, TNF-α, tumor necrosis factor-α.;VCAM, vascular cell adhesion molecule.

**Table 4:** Studies on impact of PUFA consumption on body weight.

Type of the Study	Reference	Year	Type of Intervention	Primary Outcome	Conclusions
In vitro	Di Nunzio, M. et al. [83]	2011	Supplementation of HepG2 cells with different PUFAs produced various effects on cytotoxicity, oxidation and on antioxidant defenses.	Supplementation with ARA highlighted the induction of oxidative damage, on the contrary, DHA supplementation induced an enhancement in antioxidant defenses.	Each PUFA seems to exert certain actions, on the basis of chemical structure.
Human	Summers, L.K. et al. [85]	2002	17 subjects (6 with T2DM, 6 non-obese and 5 obese without T2DM) were randomized in a crossover study to follow two 5-week periods (one period with a diet rich in saturated fatty acids and one period with a diet rich in polyunsaturated fatty acids).	Insulin sensitivity and plasma LDL cholesterol concentrations ameliorated in subjects that followed a diet rich in PUFA compared with the subjects that followed a diet rich in saturated fatty acids. The authors observed a reduction in abdominal subcutaneous fat.	These dietary patterns suggest an improvement in insulin sensitivity, reducing the risk of developing T2DM.
	Couet, C. et al. [86]	1997	Six volunteers were fed with a control diet (52% carbohydrates, 16% protein, 32% fat; no FO) *ad libitum* for 3 weeks and, 10–12 weeks later, 6 g of fats, of the same diet, were replaced with 6 g/d of FO for a further 3 weeks.	After the dietetic treatment with FO, there was observed a decrease in body fat mass and basal respiratory quotient and an increase of basal lipid oxidation.	Dietary FO causes a reduction of body fat mass and induces lipid oxidation in healthy adults.
	Huang, T. et al. [87]	2019	The authors tested interactions of ω-3 PUFA habitual consumption and overall genetic susceptibility on long-term weight change.	Food-sourced ω-3 PUFA assumption showed substantial interactions with GRS on long-term changes in body weight.	High intake of ω-3 PUFAs is related to an attenuation of genetic association with long-term weight gain.
	Vaughan, L.K. et al. [88]	2015	The authors evaluated body composition, plasma adipokines and ghrelin in 982 subjects. They investigated gene–diet interactions.	The authors observed a linkage for all obesity-related traits. They identified new regions of interest for adiponectin and body circumferences. They reported that ω-3 PUFAs are able to modify the link with obesity-related traits.	These authors speculated on the interaction between gene-obesity tract and the pathophysiology obesity.
	Casas-Agustench, P. et al. [91]	2009	29 healthy men were randomized in a crossover trial. The authors compared the thermogenic effects of 3 isocaloric sources: (i) high in polyunsaturated fatty acids from walnuts, (ii) high in monounsaturated fatty acids from olive oil and (iii) high in saturated fatty acids from fat-rich dairy products.	Five hours postprandial thermogenesis was greater after the high-polyunsaturated meal (i), and after the high-monounsaturated meal (ii) compared to the high-in-saturated meal (iii).	The thermogenic response was influenced by the fat quality, although the action on substrate oxidation or satiety was unknown.
	Lund, A.S. et al. [94]	2013	1212 healthy individuals were enrolled and the authors collected information on nutritional habits associated with different measures of body fat, and inflammatory biomarkers.	Absolute ω-3 PUFA intake presents inverse correlation with anthropometric measures of body fat and among ω-3 PUFA derivatives. In particular, only ALA was inversely associated with body fat measures.	Intake of ω-3 PUFA, in particular ALA, is positively associated with body fat.

Abbreviations: ALA, α-linolenic acid; ARA, arachidonic acid;; DHA, docosahexaenoic acid; FO, fish oil; GRS, genetic risk score; LDL, low-density lipoproteins; PUFA, polyunsaturated fatty acid;T2DM, type 2 diabetes mellitus.

**Table 5:** Studies on PUFAs and metabolic axis.

Type of the Study	Reference	Year	Type of Intervention	Primary Outcome	Conclusions
In vitro	Andrade-Vieira, R. et al. [116]	2013	In vitro tests on MCF7 and HeLaS cell lines.	ω-3 PUFA increases the activation of the Akt-mTOR-S6K1 anabolic pathway.	ω-3 PUFA supplementation can improve cellular metabolism by the promotion of anticatabolite and anabolites production.
	Perez-Matute, P. et al. [109]	2007	Male Wistar rats were fed a high-fat diet for 5 weeks: (i) with oral administration of EPA (1 g/kg) or (ii) without EPA administration.	The increase in body weight and FM was lower in the group treated with EPA. Moreover, EPA administration induced a decrease in food intake and an increase in leptin production and was able to prevent the increase in TNFα.	EPA supplementation can increase the feeling of satiety and reduce the inflammatory state induced by a high-fat diet.
	Takahashi, Y. et al. [80]	2000	4 groups of rats were fed for 21 days as follows: (i) low-fat diet with 20 g of safflower oil/kg; (ii) high-fat diet (200 g/kg) rich in ω-6 with safflower oil; (iii) high-fat diet (200 g/kg) rich in ω-3 with perilla; (iv) high-fat diet (200 g/kg) rich in ω-3 with fish oil.	The high-fat diets rich in ω-3, compared to a low-fat diet, did not increase the WAT mass, but increased BAT. Moreover, the diets rich in ω-3, reducing the expression of GLUT-4 mRNA in WAT.	In rats, the gene expression of GLUT-4 mRNA in adipose tissue by ω-3, prevents body fat accumulation and regulates glucose metabolism.
	Whitehouse, A.S. et al. [114]	2001	Evaluation of the effect of EPA administration on soleus muscle proteolysis during acute fasting in rats compared to control group (olive oil).	Significant reduction of soleus muscle proteolysis in an EPA-treated group and attenuation of the proteasome “chymotryptic-like” enzyme activity.	EPA is able to inhibit protein proteolysis during acute starvation.
Human	Madsen, E.L. et al. [103]	2009	Treatment of 68 subjects in abdominal obesity with a low-calorie diet (600–800 kcal/die) for 8 weeks followed by 36 months of randomized treatment with (i) orlistat or (ii) placebo, in association with the lifestyle intervention.	The decrease in body weight is associated with a significant reduction of IL-18, MMP-9 and leptin levels.	Long-term weight loss reduces non-traditional CV risk factors.
	Parra, D. et al. [97]	2008	Appetite monitoring in 233 volunteers during the last 2 weeks of an 8-week low-calorie diet, associated with: (i) low ω-3 intake (<260 mg/die) or (ii) high ω-3 intake (>1300 mg/die).	The evaluation of VAS reveals lower hunger in the high-ω-3 group after dinner after 120 min.	ω-3 intake modulates postprandial satiety in obese and overweight subjects during the weight loss.
	Shamsuzzaman, A.S. et al. [107]	2004	Association study between plasma leptin and CRP in 100 healthy subjects.	Strong positive and significant association between leptin and CRP, even after adjustment for age, BMI, waist-hip ratio, smoking and alcohol consumption.	The study confirms a strong correlation between metabolic and inflammatory mechanisms.
	Dimiter, V. [113]	2007	35 overweight and obese adults with metabolic syndrome were randomized into 2 groups: (i) treated with ω-3 and (ii) treated with placebo, for 3 months on a normal diet, without lifestyle changes.	Increased plasma concentrations of HDL-C and adiponectin and decreased plasma concentrations of triglycerides, HOMA-IR and CRP after ω-3 treatment.	ω-3 PUFA supplementation can improve the inflammatory status and lipid profile through adiponectin-dependent mechanisms in patients with metabolic syndrome.

Abbreviations: Akt- mTOR, protein kinase B- mammalian target of rapamycin; BAT, brown adipose tissue; BMI, body mass index;CRP, C-reactive protein; CV, cardiovascular; EPA, Eicosapentaenoic acid; FM, fat mass; GLUT-4, glucose transporter type-4; HDL-C, high density lipoprotein-cholesterol; HOMA-ir, homeostatic model assessment for insulin resistance; IL-18, interleukin-18; MMP-9, matrix metalloproteinase-9; PUFA, polyunsaturated fatty acids; TNFα, tumor necrosis factor-α; VAS, visual analogue scale WAT, white adipose tissue.

## Abbreviation List

AA	Arachidonic acid
AHA	American Heart Association
Akt	Protein kinase B
ALA	α-linoleic acid
AMI	Myocardial infarction
BAT	Brown adipose tissue
BMI	Body mass index
CAD	Caspase-activated DNase
CAT	Catalase
CNCD	Chronic non-communicable degenerative disease
CRP	C-reactive protein
CV	Cardiovascular
CVD	Cardiovascular disease
DART	Diet And Reinfarction Trial
DGLA	Dihomo-γ-linoleic acid
DHA	Docosahexaenoic acid
DPA	Docosapentaenoic acid
eNOS	Endothelial nitric oxide synthase
EPA	Eicosapentaenoic acid
FAs	Fatty acids
FM	Fat mass
FSH	Follicle-stimulating hormone
Gal-3	Galectin-3
GISSI	Gruppo Italiano per lo Studio della Streptochinasi nell’Infarto
GLA	γ-linoleic acid
GnRH	Gonadotropin-Releasing Hormone
HC	Hip circumference
HDL	High-density lipoprotein
HOMA-IR	Homeostatic model assessment-insulin resistance
HT	High triglyceride
IL	Interleukin
LA	Linoleic acid
LDL	Low-density lipoprotein
LH	Luteinizing hormone
LTS	Leucotriens
LXS	Lipoxines
MIP	Macrophage inflammatory protein
MOSH	Male obesity secondary hypogonadism
mTOR	Mammalian target of rapamycin
MUFAs	Monounsaturated fatty acids
NF-κB	Nuclear factor kappa-light-chain-enhancer of activated B cells
PG	Prostaglandins
PPARα	Peroxisome proliferator- activated receptor α
PUFA	Polyunsaturated fatty acid
S6K1	Ribosomal protein S6 kinase beta-1
SFAs	Saturated fatty acids
SNPs	Single-nucleotide polymorphism
SOD	Superoxide dismutase
STAT3	Signal transducer and activator of transcription 3
T	Testosterone
T2DM	Type 2 diabetes mellitus
TG	Triglycerides
TNF-α	Tumor necrosis factor- α
Tx	Thromboxanes
VCAM-1	Vascular cell adhesion molecule-1
VHT	Very high triglyceride
WAT	White adipose tissue
WC	Waist circumference
WHO	World Health Organization
